# Remodeling of Mitochondria–Endoplasmic Reticulum Contact Sites Accompanies LUHMES Differentiation

**DOI:** 10.3390/biom15010126

**Published:** 2025-01-14

**Authors:** Emad Norouzi Esfahani, Tomas Knedlik, Sang Hun Shin, Ana Paula Magalhães Rebelo, Agnese De Mario, Caterina Vianello, Luca Persano, Elena Rampazzo, Paolo Edomi, Camilla Bean, Dario Brunetti, Luca Scorrano, Samuele Greco, Marco Gerdol, Marta Giacomello

**Affiliations:** 1 Department of Biology, University of Padua, 35131 Padua, Italy; emad.norouziesfahani@phd.unipd.it (E.N.E.); tomas.knedlik@unipd.it (T.K.); 2declan@gmail.com (S.H.S.); paularebelo94@gmail.com (A.P.M.R.); caterina.vianello@unipd.it (C.V.); luca.scorrano@unipd.it (L.S.); 2 Department of Biomedical Science, University of Padua, 35131 Padua, Italy; agnese.demario@unipd.it; 3 Department of Women’s and Children’s Health, University of Padua, 35128 Padua, Italy; luca.persano@unipd.it (L.P.); elena.rampazzo@unipd.it (E.R.); 4 Pediatric Research Institute, Città della Speranza Foundation, 35127 Padua, Italy; 5 Department of Life Sciences, University of Trieste, 34127 Trieste, Italy; edomi@units.it (P.E.); samuele.greco@units.it (S.G.); mgerdol@units.it (M.G.); 6 Department of Medicine, University of Udine, 33100 Udine, Italy; camilla.bean@uniud.it; 7 Unit of Medical Genetics and Neurogenetics, Fondazione IRCCS Istituto Neurologico “C. Besta”, 20126 Milan, Italy; dario.brunetti@unimi.it; 8 Department of Clinical Sciences and Community Health, University of Milan, 20122 Milan, Italy; 9 Veneto Institute of Molecular Medicine, 35129 Padua, Italy

**Keywords:** Neural precursor cells, LUHMES, mitochondria, mitochondria–ER contact sites, MERCs, differentially expressed genes

## Abstract

Neural progenitor cells (NPCs) are often used to study the subcellular mechanisms underlying differentiation into neurons in vitro. Works published to date have focused on the pathways that distinguish undifferentiated NPCs from mature neurons, neglecting the earlier and intermediate stages of this process. Current evidence suggests that mitochondria interaction with the ER is fundamental to a wide range of intracellular processes. However, it is not clear whether and how the mitochondria–ER interactions differ between NPCs and their differentiated counterparts. Here we take advantage of the widely used NPC line LUHMES to provide hints on the mitochondrial dynamic trait changes that occur during the first stage of their maturation into dopaminergic-like neurons. We observed that the morphology of mitochondria, their interaction with the ER, and the expression of several mitochondria–ER contact site resident proteins change, which suggests the potential contribution of mitochondria dynamics to NPC differentiation. Further studies will be needed to explore in depth these changes, and their functional outcomes, which may be relevant to the scientific community focusing on embryonic neurogenesis and developmental neurotoxicity.

## 1. Introduction

Neurogenesis is a complex series of events that begins with the commitment of neural progenitor cells (NPCs) derived from neural stem cells (NSCs) and ends with their differentiation into neurons [1]. This highly regulated process relies on the coordination of intrinsic and extrinsic molecular signaling pathways that converge to direct NPCs toward the proper cell fate. The proliferation and differentiation processes of NPCs are fine-tuned by transcriptional factors and other pathways [2,3]. Moreover, maturation into neurons involves cell cycle arrest and cessation of proliferation.

During differentiation of NPCs, genes coding for synaptic proteins (e.g., glutamate ionotropic receptor NMDA type subunit 1, GRIN1; Discs large MAGUK scaffold protein 4, DLG4) and dopaminergic markers (e.g., tyrosine hydroxylase, TH; dopamine D2-receptor, DRD2; dopamine transporter, SLC6A3) are upregulated [4]. Besides the obvious morphological changes (e.g., neuritogenesis), differentiation is accompanied by a strong alteration in the metabolic phenotype [5,6].

In particular, proliferating cells have a higher demand for glucose and an increased glycolytic rate, which contributes to the generation of ATP [7]. In contrast, adult neurons metabolize glucose to CO_2_ through oxidative phosphorylation via the tricarboxylic acid cycle [8]. A number of metabolic changes occur during NPC differentiation, from glycolysis to oxidative phosphorylation [6,9,10]. While glycolysis is involved in the early phases of differentiation, mitochondrial bioenergetics is required for later stages, such as dendritic sprouting [11].

The structure of mitochondria is crucial for their bioenergetic output; in the context of neurogenesis, it has been shown that their morphology accompanies and contributes to the fate of NSCs [3,12]. As NSCs begin to differentiate in the adult mouse hippocampus, mitochondrial biogenesis and length increase, becoming significantly longer in dentate granule neurons [13]. In addition, it has been shown that, in the mouse embryonic cortex, mitochondria fragmentation promotes the differentiation of NPCs into neurons [14]. The above-mentioned processes suggest a potential connection between mitochondrial dynamics and NPC differentiation.

It is now largely recognized that the crosstalk between mitochondria and other organelles substantially controls mitochondrial functions. This inter-organelle interaction occurs at points in which the surfaces of the two compartments are in high proximity, which are known as “membrane contact sites” [15]. For example, mitochondria–endoplasmic reticulum contact sites (MERCs), separated typically by a gap of 10–80 nm, help to control calcium and lipid homeostasis to regulate mitochondrial dynamics, and they also participate in autophagy initiation [15]. Interestingly, MERCs have been shown to be involved in cardiomyocyte differentiation [16]. However, whether and how contact sites between mitochondria and other organelles contribute to NPC differentiation remains to be determined.

Therefore, we set out to investigate whether and how changes in MERCs are involved in the differentiation of the NPC model cell line LUHMES into post-mitotic dopaminergic neurons. We found that the expression levels of several MERC resident proteins decrease in parallel with those of proteins previously associated with NPC physiology, suggesting a potential link between MERCs and NPC differentiation.

## 2. Materials and Methods

### 2.1. Cell Culture

LUHMES were grown as previously reported [17]. Briefly, cells were cultured in flasks coated with poly-L-ornithine (0.1 mg/mL; 4 °C, overnight; Sigma-Aldrich, St. Louis, MO, USA) in a DMEM/F12 medium (Sigma-Aldrich) with 1% N2-supplement (#17502048 Thermo Fisher Scientific, Waltham, MA, USA), 2 mM L-Glutamine (Thermo Fisher Scientific) and a 0.04 μg/mL human basic fibroblast growth factor (bFGF, Gibco, #13256-029, Thermo Fisher Scientific). For differentiation into neurons, LUHMES were plated at a density of 100,000 cells per cm^2^ in cell culture dishes previously coated with poly-L-ornithine (50 μg/mL; 4 °C, overnight; Sigma-Aldrich) and human fibronectin (1 μg/mL, 37 °C, overnight; Sigma-Aldrich). −24 h after seeding, differentiation was induced by using DMEM/F12 with 1% N2-supplement, 1 μg/mL tetracycline (Sigma-Aldrich), 1 mM dibutyryl cAMP (Sigma-Aldrich), and 2 ng/mL glial cell-derived neurotrophic factor (GDNF) (Sigma-Aldrich, #G1777).

### 2.2. Mouse Brain Fractionation

Mouse brains were fractionated according to the previously published protocol [18], starting with the pre-cooling of the buffers and the homogenizer (with a Teflon pestle). The isolated mouse brains were halved, with one half used to obtain mitochondria-associated ER membranes (MAMs). For their extraction, half-brains were homogenized in 225 mM D-mannitol, 75 mM sucrose, 30 mM Tris/HCl pH 7.4, 1 mM EGTA, and 10 mM HEPES (“isolation buffer”; IBB). Brains were placed in 50 mL centrifuge tubes, rinsed with PBS containing 10 mM EDTA, minced, and trypsinized for 30 min at 4 °C. Samples were centrifuged at 700× *g* for 10 min at 4 °C and resuspended in ice-cold IBB. The samples were then homogenized using a glass–Teflon homogenizer (10 strokes at 1500 rpm on ice), centrifuged at 800× *g* for 5 min at 4 °C, and the supernatant was collected. After further centrifugation at 800× *g* for 5 min at 4 °C, the lysate fractions were obtained, while the pellet was resuspended in the IBB (obtaining the post-nuclear supernatant fraction). Further centrifugation allowed the separation of the mitochondrial pellet from the cytosolic fraction, which was further centrifuged at 20,000× *g* for 30 min at 4 °C to isolate microsomes and ER fractions. The mitochondria were gently resuspended in IBB and centrifuged twice at 8000× *g* for 10 min at 4 °C to yield a crude mitochondrial fraction. Finally, the pure mitochondrial fraction was obtained by ultracentrifugation through a Percoll gradient (225 mM D-mannitol, 25 mM HEPES/KOH pH 7.4, 1 mM EGTA, 30% (*v*/*v*) Percoll), using a swing-out rotor (95,000× *g* for 30 min at 4 °C, with “slow brake” mode). At this stage, the MAMs fraction appeared as a dense white band in the middle of the gradient, while the pure mitochondrial fraction appeared as a yellow band near the bottom of the tube.

Animal housing and all the experimental procedures were authorized by the Italian Ministry of Health (approved protocols: 383-2015-PR 2015-05-19).

### 2.3. Library Preparation and mRNA Sequencing

LUHMES cells were lysed, and RNA was purified with Trizol^®^ (Thermo Fisher Scientific, #15596018) according to the manufacturer’s protocol. Sample purity and concentration were checked using a NanoDrop spectrophotometer (ND 1000, Thermo Fisher Scientific). Libraries were prepared with the QuantSeq 3’ mRNA-seq Library Prep Kit (Lexogen, Vienna, Austria) and oligo(dT) primers. External barcodes were introduced during the PCR amplification step. Library quantification and quality control were performed using a fluorometer (Qubit, Thermo Fisher Scientific) and Bioanalyzer 2100 (Agilent, Santa Clara, CA, USA). NGS reads were generated against the poly(A) tail. Sequencing was performed on an Illumina NextSeq500 (Ilumina, San Diego, CA, USA) instrument to generate a minimum of 5 × 10^6^ reads (75 bp SE) for each sample. Raw data were deposited in the Sequence Read Archive (SRA) database (Bioproject #PRJNA1175585).

### 2.4. Analysis of RNA-Seq Data

The quality of the sequencing reads was assessed with FastQC v0.11.9 [19], plus MultiQC v1.6 [20], then the reads were trimmed with fastp v0.20.1 by removing: (i) 15 nt at 5’, (ii) 2 nt at 3’; (iii) reads containing “N” characters; (iv) heading and trailing homopolymers; and (v) heading and trailing low-quality portions. Only trimmed reads with a length of at least 35 nt were retained. Trimmed reads were mapped to the human reference genome GRCh38.p10 using the RNA-seq analysis tool in CLC Genomics Workbench (Qiagen, Hilden, Germany) with default parameters, except for a similarity fraction of 0.95 and a length fraction of 0.9. A first check of coherence between samples was performed by visual inspection of Principal Component Analysis (PCA) and Multi-Dimensional Scaling (MDS) two-dimensional scatter plots. Differential gene expression analysis was carried out with the edgeR package [21]; briefly, genes with low counts were discarded (filterByExpr function), then expression values were normalized, and a Generalized Linear Model (GLM) was fitted on the data. All possible comparisons between sample groups were then evaluated, defining as differentially expressed genes (DEGs) those showing an FDR-corrected *p*-value lower than 0.05. Functional Enrichment Analysis was performed by gene ontology (GO) annotations. Specifically, a hypergeometric test was applied to the DEG sets (also splitting up- and down-regulated DEGs) (41) using only expressed genes as “universe” (i.e., those that were kept by the FilterByExpr function) to avoid biases [22].

### 2.5. Expression Pattern Analysis

The expression values of the DEGs across all the time points were converted to per-gene z-scores (calculated by subtracting the mean expression and dividing by the standard deviation for each gene) and clustered with the BIRCH algorithm (threshold = 0.75) to identify groups of genes displaying similar temporal expression patterns [23]. Clusters containing more than 50 DEGs were subjected to enrichment analysis, and interaction networks were built as undirected graphs for each cluster using the BIOGRID [24] database, focusing only on high-throughput evidence. Moreover, DEGs were also annotated based on their mitochondrial localization using the MitoCarta [25] database.

### 2.6. Western Blotting

Cells were lysed in an ice-cold RIPA buffer containing a protease inhibitor cocktail (Thermo Fisher Scientific, #A32955). Samples were then centrifuged at 16,000× *g* for 20 min at 4 °C, and the protein content of the supernatant was measured with the Bradford reagent (Bio-Rad, #5000006, Hercules, CA, USA). After heating at 70 °C for 10 min, 30 μg of proteins was loaded onto precast gels (ExpressPlus™ PAGE Gel, Genscript 4–12% or 4–20%, Piscataway, NJ, USA). After electrophoresis in Tris-MOPS buffer (Genscript, #M00138), the proteins were blotted onto a PVDF membrane (#IPVH85R EMD Millipore, Merck, MA, USA). Blocking was performed for 1 h at room temperature in Tris-buffered saline with 5% milk (Sigma Aldrich, #70166) containing 0.1% Tween 20. The membrane was incubated with primary antibodies overnight at 4 °C. Membranes were washed with Tris-buffered saline and then incubated with HRP-conjugated anti-rabbit secondary antibody (1:4000; Cell Signalling, #7074-P2, Danvers, MA, USA) and HRP-conjugated anti-mouse secondary antibody (1:2000 or 1:4000; Cell Signalling, #7076-P2) for 1 h at room temperature. The signal was visualized using the enhanced chemiluminescence kit Luminata Forte Western HRP (Millipore Sigma WBLUF0500, Burlington, MA, USA) according to the manufacturer’s instructions and acquired using ImageQuant LAS 4000 Mini (GE Healthcare, Chicago, IL, USA) or the ChemiDoc MP Imaging System (Bio-Rad).

Densitometry analysis was performed using Image Studio Lite (Ver.5.2) software. In brief, a small rectangular region of interest around each band was drawn and the corresponding intensity was measured. The intensity of each band was subtracted from its local background intensity and normalized to day 0. One-way ANOVA was used for statistical significance, and the handling of the data was performed in GraphPad Prism 10.

### 2.7. Transmission Electron Microscopy

LUHMES cells were seeded into 24-well plates and fixed after 48 h cells as previously described [26]. Briefly, samples were initially fixed overnight at 4 °C with 2.5% glutaraldehyde in 0.1 M sodium cacodylate buffer pH 7.4, followed by secondary fixation with 0.1 M sodium cacodylate buffer containing 1% osmium tetroxide and 1% potassium ferrocyanide for 1 h at 4 °C. After three washes, samples were dehydrated and embedded in an epoxy resin (Sigma-Aldrich). Sections of 60–70 nm thickness were cut with a Leica Ultracut EM UC7 ultramicrotome, counterstained with uranyl acetate and lead citrate, and observed with Tecnai G2 (FEI) transmission electron microscope operating at 100 kV. Images were captured with a Veleta (Olympus Soft Imaging System, Olympus, Münster, Germany) digital camera. TEM images and experiments were performed at the Electron Microscopy Facility of the University of Padua.

### 2.8. TEM Image Analysis

TEM images were imported into Fiji 1.49m software where image scaling was standardized using the “Set Scale” command to ensure consistent scaling across all images. To quantify the mitochondrial perimeter and the dimensions of mitochondrial-endoplasmic reticulum contact sites (MERCs), the “Freehand” and “Straight Line” tools in Fiji were employed. Specifically, mitochondria and ER membranes visible on TEM images were marked with lines drawn using the “Freehand” tool. These annotations defined the regions of interest (ROIs) and the lengths of the lines (i.e., mitochondrial perimeter and MERC dimensions) were recorded and saved in the ROI Manager. These measurements were then exported to an Excel spreadsheet for data collation and subsequent statistical analysis. This approach ensured accurate and reproducible quantification of MERC structures across all images. Details of the statistical procedures used for data analysis are provided in the following section “Statistical Analysis”.

To assess the distribution of mitochondria–ER contact widths, we performed a frequency distribution analysis. First, the width of all mitochondria–ER contact sites was measured for all cells across all experimental days using TEM image analysis (see the previous paragraph). Next, each measured contact was categorized into one of three predefined groups based on its width (0–30 nm, 30–60 nm, and 60–90 nm). For each cell, the total number of contact sites across all three groups was calculated. The percentage of contact sites in each category was then determined for each cell by dividing the number of contacts in a given category by the total number of contacts in that cell and multiplying by 100.

### 2.9. Statistical Analysis

All data are expressed as mean ± SEM unless otherwise stated. At least three independent experiments were carried out unless otherwise specified. Statistical tests used are indicated in the figure legends. Definitive outliers were removed using Grubb’s method with an alpha equal to 0.0001. Normality tests were performed in GraphPad Prism 10 using the following methods: the D’Agostino–Pearson omnibus normality test, Anderson–Darling test, Shapiro–Wilk normality test, and the Kolmogorov–Smirnov normality test. Depending on the results of normality tests, parametric (ordinary one-way ANOVA) or non-parametric (Kruskal–Wallis test) statistical tests were used for normally distributed or skewed data, respectively. All statistical tests and *p*-value calculations were performed automatically using GraphPad Prism 10. Significance was adjusted at *p* < 0.05.

## 3. Results

### 3.1. MERC and Mitochondria Morphology Changes Accompany the Differentiation of LUHMES into Post-Mitotic Dopaminergic Neurons

**Figure 1:**
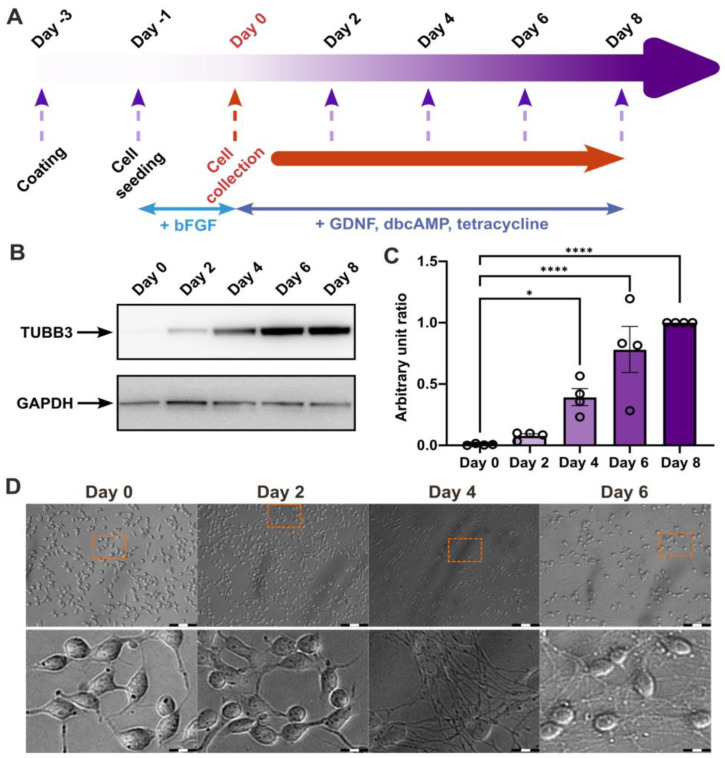
Differentiation of LUHMES cells. (**A**) Scheme of LUHMES differentiation: at day 0, cells were cultured in a medium supplemented with GDNF, dcAMP, and tetracycline, and cell samples were collected every 2 days, up to 8 days of differentiation. The orange arrow indicates the cell collection phase. (**B**) Western blot analysis of β-tubulin III expression. (**C**) Quantification of β-tubulin III expression using densitometry (*n* = 4 independent experiments; normalized to day 8). (**D**) Differentiation of LUHMES cells was visualized using bright-field microscopy; scale bars: 100 μm (up) and 15 μm (bottom). Statistical significance was determined using ordinary One-way ANOVA test. Statistical significance: * *p*-value < 0.05, **** *p*-value < 0.0001. Western blot original images are in the supplementary materials.

To investigate the potential role of MERCs in NPC differentiation, we employed the well-established human neural precursor cell model LUHMES and the previously described protocol (Figure 1A), which has been shown to allow their differentiation into post-mitotic neurons with dopaminergic properties [4]. First, we confirmed the correct differentiation of LUHMES into neuronal cells by quantifying the neuronal marker β-tubulin III with Western blots (Figure 1B,C) and imaging the cells at different time points (Figure 1D), i.e., day 0, day 2, day 4, day 6, and day 8. In line with the increasing expression of β-tubulin III, LUHMES cells show substantial morphological changes along differentiation from stem-like to neuronal morphology with long axons, as reported before [4].

**Figure 2:**
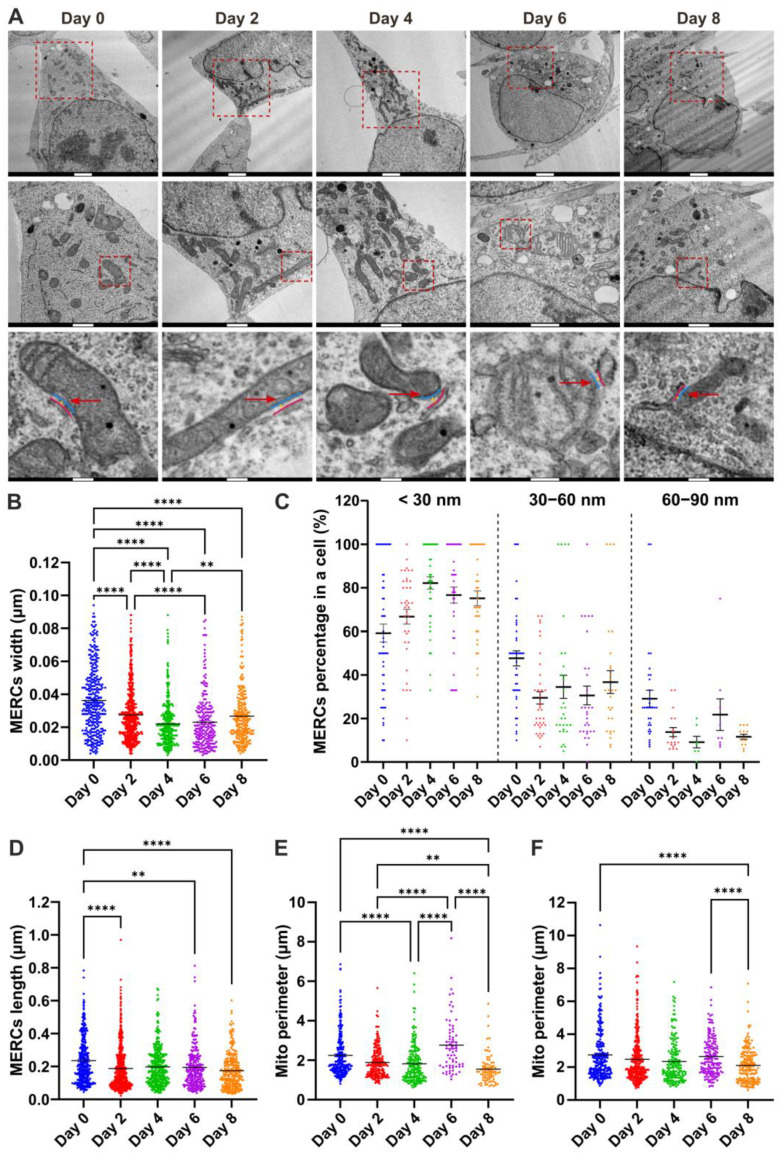
Analysis of MERC structure in differentiating LUHMES cells via TEM. (**A**) Representative TEM pictures of LUHMES cells at the indicated timepoint; scale bars: 2 μm (up), 1 μm (middle); 0.2 μm (bottom). Representative MERCs are indicated by red arrows; the ER membrane is marked by a magenta line, the outer mitochondrial membrane by a cyan line, and the MERC width by a yellow line. (**B**) Quantification of the MERC width. (**C**) Histogram of the distribution of the MERC width along differentiation. (**D**) Quantification of the MERC length. (**E**) Quantification of the perimeter of mitochondria in contact with the ER. (**F**) Quantification of the perimeter of mitochondria not engaged in contact sites with the ER. Error bars represent the standard error of the mean (SEM). Statistical significance was determined using the Kruskal–Wallis test. Statistical significance: ** *p*-value < 0.01, **** *p*-value < 0.0001.

Subsequently, to assess whether MERC changes accompany the differentiation of NPCs into dopaminergic neurons, we analyzed in detail the structure of MERCs in transmission electron microscopy (TEM) images (Figure 2A). We considered a range of parameters, including the number of contacts, their lengths, and their widths, as these could reflect diverse types and activities of MERCs [15,26].

We found that the width of MERCs shortened during differentiation, with a sharp decrease from day 0 to day 4 (Figure 2B). In parallel, the frequency distribution of MERC width (describing the percentage of contact sites within specific intervals of width in a cell) was markedly elevated in MERCs with a width below 30 nm, and, conversely, reduced in those with a greater width. Then, we determined the frequency distribution of the MERC width within each individual cell. This ensures that the data reflect the variability in contact site widths across different cells. We found that from day 4 to day 8, the width of the MERCs and their frequency distribution tended to revert, indicating the dynamic nature of the MERC structure during differentiation. (Figure 2C). The lengths of MERCs also decreased during differentiation, especially from day 0 to day 2 (Figure 2D). Finally, we investigated whether differentiation into neurons was also accompanied by changes in the mitochondrial morphology, as previously observed [3,13]. Therefore, we quantified the perimeter of mitochondria either involved in MERCs or not engaged in these sites. As shown in Figure 2E, the perimeter of mitochondria involved in MERCs tends to decrease as cells mature into neurons, with a notable increase in the perimeter at day 6. In addition, a similar decreasing trend was observed in the perimeter of mitochondria not involved in MERCs (Figure 2F). In this study, we aimed to investigate the earliest stages of differentiation in MERCs; therefore, we focused our attention on the changes that occurred from day 0 to day 2, where both the length and width of the contacts appeared to differ.

Overall, these results indicate that LUHMES differentiation is accompanied by changes in MERCs, which are independent of the variations in mitochondrial morphology.

### 3.2. Genes Associated with Cell Cycle and Mitochondria Dynamics Are Differentially Expressed During Neuronal Differentiation of LUHMES Cells

**Figure 3:**
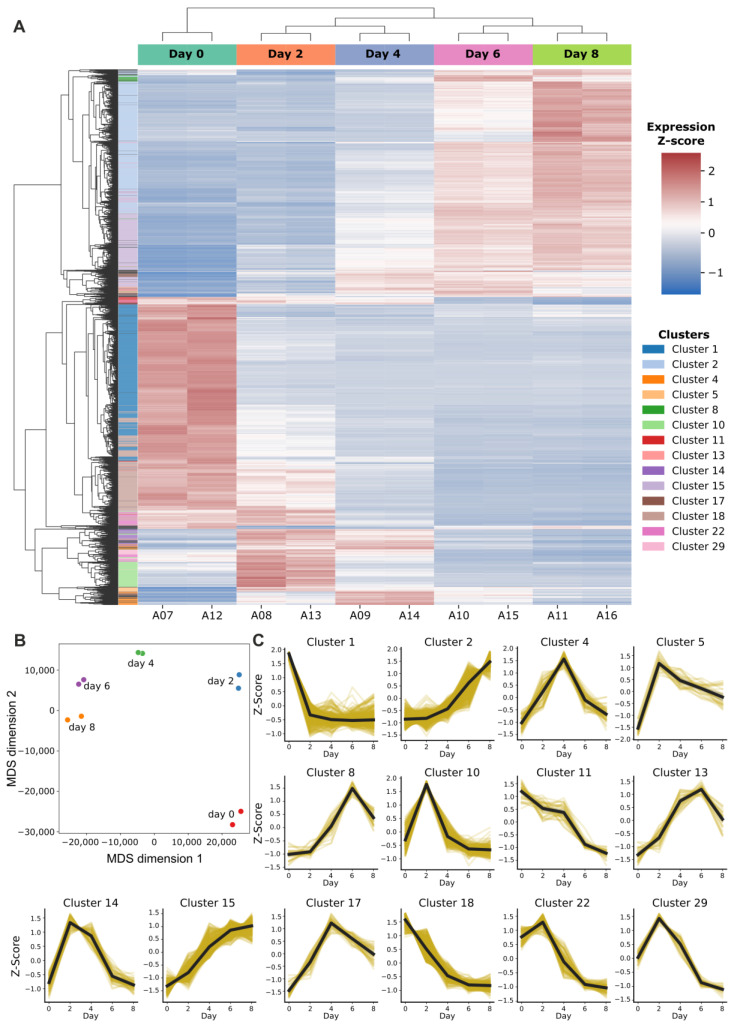
Differentially expressed genes along differentiation of LUHMES cells. (**A**) Heatmap of the identified differentially expressed genes (DEGs). A total of 3083 DEGs were identified using a multiple comparison analysis with Bonferroni correction, *p*-value = 0.005. The Z-score was calculated based on log(cpm) values scaled by rows. (**B**) Multidimensional scaling (MDS) plot showing sample dissimilarity based on pairwise distances, with similar samples clustering together (**C**) Birch clustering showing the patterns of change in gene expression; 30 clusters were identified, of which 14 clusters, shown here, contained more than 50 DEGs. Birch threshold: 0.75.

To gain molecular insights into the observed changes in MERCs and mitochondrial morphology, we performed a transcriptomic analysis. We collected samples at each time point and analyzed their gene expression profiles employing RNA sequencing. By comparing expression levels between each time point and the undifferentiated state, we identified 3083 non-redundant differentially expressed genes (DEGs) with a corrected *p*-value threshold of 0.005 (Figure 3A, Table S1). The diversity between the differentiation stages could be clearly recognized at the transcriptional level since both replicates clustered in a distinct group separated from each other (Figure 3B). Of note, cells at day 0 displayed the most distinct gene expression profiles compared to other time points, which reflects the undifferentiated state of the cells as neuronal progenitor cells before the onset of differentiation (Figure 3B). The differentiation of the cells into dopaminergic-like neurons was confirmed by the identification of markers of dopaminergic neurons among the DEGs, including tyrosinase, LIM homeobox transcription factor 1 alpha/beta, the forkhead box transcription factors FOXA1/2/3, and engrailed homeobox 1 and orthodenticle homeobox 2 [27].

To find patterns of change in gene expression, regardless of their absolute expression values, we used the Birch clustering approach. After the identification of 14 clusters with more than 50 DEGs, we performed gene ontology (GO) enrichment analyses in clusters comprising a minimum of 50 DEGs (Figure 3C). Among the over-represented GO terms associated with these clusters (Table S2), we discovered several expected functional categories, such as axon guidance, neuron projection and regulation of dopamine secretion (cluster 2), regulation of apoptosis (cluster 10), and cell differentiation (cluster 14). One of the most enriched clusters, in terms of gene number, is cluster 15, which includes genes that are progressively upregulated and are involved in neuronal differentiation. In cluster 15 we also found genes associated with extracellular matrix remodeling and cytoskeletal dynamics, highlighting the importance of these processes in differentiation, as previously shown [28]. The observed pattern of gene expression, whether up- or down-regulated, was consistent with the expected functional GO terms, suggesting a reliable clustering of gene expression.

Notably, cluster 10 was particularly enriched in transcription factors (TFs), including *TLX2*, *GATA2*, *FOXD1*, *FOXP2*, *CEBPD*, *MAFA*, *NFKB2*, *ETS1*, *SOX1*, *SOX9*, and *JUNB*. Among our DEGs, we found 532 genes known to be targets of these TFs, which also show variable expression along differentiation (Table S3). The pattern of cluster 10, with a peak at day 2 followed by a sharp decline at day 4, suggests that TF activity at day 2 is one of the hallmarks of transcriptional reprogramming during LUHMES differentiation. We observed a similar pattern in clusters 14, 22, and 29 enriched in GO terms related to positive regulation of transcription by RNA polymerase II and cell differentiation. Collectively, this suggests that major transcriptional changes occur within the first two days of differentiation, where multiple pathways are orchestrated to promote this process.

As we aimed to define the contribution of MERCs to neuronal cell differentiation, we primarily searched for mitochondrial and MERC genes in different clusters. We performed a binomial test to check if the expected/observed numbers of mitochondrial genes in each cluster significantly differ. Clusters 1 and 18 displayed a high enrichment of mitochondrial genes that were downregulated along differentiation, with cluster 1 containing the highest number of DEGs coding for mitochondrial proteins (Table 1). In particular, among the DEGs, we identified 13 genes coding for MERC-resident proteins, mostly belonging to cluster 1 (Table 2).

**Figure 4:**
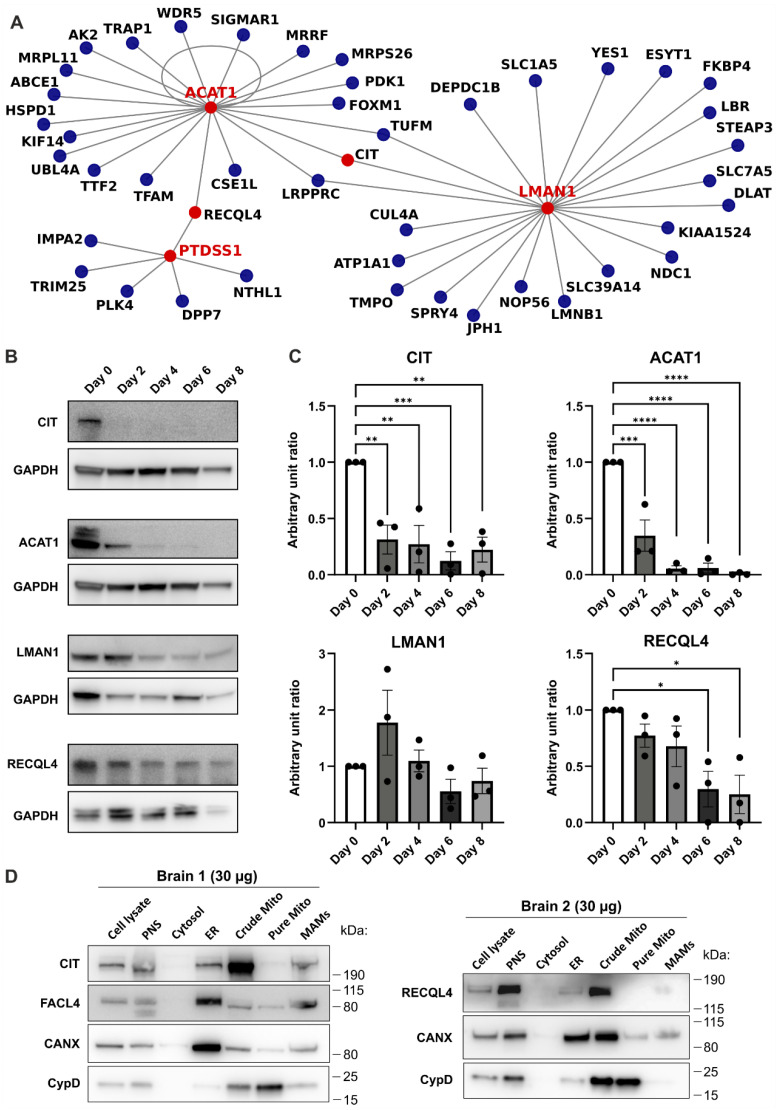
MERC resident proteins and their protein interactors among DEGs. (**A**) Cluster 1 MERC resident proteins and their known protein interactors among DEGs using the BIOGRID database. (**B**) Western blot of the MERC resident proteins and their interactors, CIT and RECQL4. GAPDH was used as an internal control. (**C**) Densitometry analysis of Western blots. A small region of interest was drawn around each band, and the band intensity was acquired after background subtraction. Ordinary one-way ANOVA was used for statistical significance. Statistical significance: * *p* value < 0.05, ** *p* value < 0.01, *** *p* value < 0.001, **** *p* value < 0.0001. (**D**) Western blots of mouse brain subcellular fractions; 30 μg of protein were loaded. CANX: Calnexin, FACL4: Acyl-CoA Synthetase Long-Chain Family Member 4; PNS: post-nuclear supernatant fraction; MAMs: mitochondria-associated membranes fraction. Western blot original images are in the supplementary materials.

Therefore, we decided to analyze the protein–protein interactions between the MERC-resident proteins and DEGs belonging to cluster 1 using the BIOGRID database (Figure 4). This could potentially reveal novel interactors with proteins resident in the MERCs, as they show the same pattern along the differentiation, as well as key factors involved in the process. Indeed, among the DEGs belonging to cluster 1, we identified a small network of proteins including three proteins residing at MERCs: LMAN1 (lectin mannose-binding 1), ACAT1 (acetyl-CoA acetyltransferase), and PTDSS1 (phosphatidylserine synthase 1) (Figure 4A). ERO1A (endoplasmic reticulum oxidoreductase 1 alpha) had no interaction among DEGs, and DGAT2 (diacylglycerol O-acyltransferase 2) interacted with only one protein (i.e., DDX39A); therefore we focused on the three proteins with the highest interacting network among DEGs.

According to the BIOGRID protein–protein interaction analysis (Figure 4A), LMAN1 and ACAT1 had three common interactors: CIT (citron Rho-interacting kinase), LRPPRC (Leucine-rich PPR motif-containing protein, mitochondrial), and TUFM (elongation factor Tu, mitochondrial). While LRPPRC and TUFM had been previously found in mitochondria or the outer mitochondrial membrane [29,30], CIT appears to be located in the nucleus [31]. As we aimed to identify new interactions within MERCs, we focused on the two proteins that connected the three well-established MERC proteins (i.e., LMAN1, ACAT1, PTDSS1), namely ATP-dependent DNA helicase Q4 (RECQL4) and CIT. First, using Western blot analysis of the collected LUHMES cell samples, we confirmed that ACAT1, and its two potential interactors CIT and RECQL4, were indeed downregulated during LUHMES differentiation (Figure 4B). The Western blots were quantified by densitometry analysis to measure relative protein levels (Figure 4C). Then, we investigated their localization through the Western blot analysis of subcellular fractions obtained from mouse brains. As shown in Figure 4D, RECQL4 and CIT could be retrieved in the crude mitochondria and mitochondria-associated membranes (MAMs) but not in the pure mitochondria fractions. As faint bands were also detectable in the PNS and ER fractions, further experiments will be necessary to confirm the multiple subcellular localization of RECQL4 and CIT.

## 4. Discussion

The goal of this work was to describe changes in MERC structure during the differentiation of the neuronal precursor cell line LUHMES, which can differentiate into post-mitotic dopaminergic neurons within 6–8 days. While the conversion of neural stem cells into neurons has been linked to the remodeling of mitochondria, the role of their contact sites with other organelles, such as the ER, in this process remains unknown [3,12].

Our experiments show that the structure of MERCs changes from the onset of differentiation. MERCs are characterized by several well-defined features, including the width of the cleft that separates the two organelles and the length of the juxtaposition of their surfaces [15]. These parameters determine the functionality of the MERCs and can vary either together or individually, depending on the needs of the cells and the processes that need to be regulated [26,32]. In our cell model, both parameters changed during differentiation: we observed an increase in organelle proximity and a decrease in contact site length. The alterations observed in MERCs appear to be independent of any variations in mitochondrial dimensions. This phenomenon was also observed in other contact sites. It was reported that the perimeter of lipid droplets (LDs) decreased during the aging process, although the percentage of LD–mitochondrial contacts covering them increased significantly [33]. In brown adipocytes, the mitochondrial volume increased fivefold following the knockout of the SEL1 protein, with a concomitant increase in the number of MERCs as well as the proximity between the two organelles [34]. These examples point to the fact that organelles and contact sites play different roles in cell physiology. For example, it has been shown that increasing the volume of mitochondria fosters ATP production [35,36]; this could be a compensatory mechanism to cope with an increased MERC distance, which in turn leads to a reduction in mitochondria Ca^2^⁺ uptake and a decrease in Ca²⁺-induced activation of ATP production. As MERCs are a fundamental platform to shape cell metabolism [37], these changes might be necessary to sustain the metabolic reprogramming that accompanies the transition from NPCs to neurons [6,9]. For example, in vitro differentiation of NPCs (derived from human induced pluripotent stem cells) into dopaminergic neurons has been shown to be accompanied by an increase in glycolysis and activation of the Krebs cycle [38], and differentiation of primary cortical neurons has been associated with an increase in glucose metabolism, mitochondrial biogenesis, and glutaminolysis. It seems plausible that different types of neurons may require the activation or inhibition of various metabolic pathways to achieve full differentiation. Our TEM image analysis also suggests that mitochondria morphology may change differently in mitochondria that are engaged in MERCs and those not engaged. Indeed we found a significant decrease in the perimeter of the mitochondria engaged in MERCs, suggesting fragmentation of these organelles, which has been proposed to promote neuronal fate [12]. These findings pave the way for further investigation, as they suggest the presence of organelle subpopulation with distinct morphological trends, which may also be metabolically distinct [39].

The occurrence of changes in mitochondrial dynamics has been further corroborated by an RNA sequencing analysis that highlighted the presence of 185 mitochondrial genes among the DEGs, of which 13 are well-known genes coding for MERC proteins. Additionally, three of these genes were identified as part of an interaction network (i.e., *ACAT1, PTDSS1,* and *LMAN1*), with two genes that have never been previously associated with MERCs (i.e., *RECQL4* and *CIT*). *ACAT1* codes for acyl-coenzyme-cholesterol acyltransferase 1, which converts cholesterol to cholesteryl esters for lipid storage [40]; *PTDSS1* encodes phosphatidylserine synthase, a key enzyme in the phosphatidylserine biosynthetic pathway [41]; and *LMAN1* codes for the endoplasmic reticulum-Golgi intermediate compartment protein-53 (ERGIC-53, also known as lectin, mannose-binding 1), which cycles between the ER, ER-Golgi intermediate compartment (ERGIC), and cis-Golgi [42,43,44]. The potential contribution of these proteins to neural cell differentiation has never been investigated, and only a limited amount of evidence suggests that they might be involved in this process. For example, it has been recently described that silencing ACAT1 promotes the differentiation of the neuroblastoma cell line SH-SY5Y [45]. PTDSS1 may indirectly support neuronal differentiation by activating phosphoinositide-3-kinase (PI3K)/AKT and Ras/Raf-mediated signaling pathways [46,47]. The potential roles of LMAN1 in MERC-related functions as well as in NPCs differentiation remain to be elucidated.

Unexpectedly, our bioinformatic protein–protein interaction analysis suggested an interaction between these molecules and CIT (a cytosolic RhoA-binding Ser/Thr kinase that regulates midbody formation, ensuring proper completion of cytokinesis in neuronal and spermatogenic precursors [48,49,50]) and RECQL4 (a DNA-damage-responsive helicase catalyzing DNA strand annealing). None of these proteins have been previously found at MERCs; however, RECQL4 has been observed in the nucleus, nucleolus, cytoplasm, and recently also in mitochondria [51,52,53]. Indeed, its localization can vary after exposure to oxidative stressors or agents inducing DNA double-strand breaks [54,55,56,57,58].

In addition, several pieces of evidence point to their involvement in NPC differentiation. Firstly, CIT was expressed in our cell models prior to differentiation (day 0), in line with its well-established role in NPC cytokinesis [59]. Its loss has been associated with a sharp reduction in the number of dividing cells; consequently, the downregulation of CIT at both mRNA and protein levels may favor the post-mitotic differentiation of LUHMES, a hypothesis that requires further investigation [59]. Secondly, RECQL4 overexpression has been observed to inhibit neural differentiation by forcing the localization of p53 at mitochondria and hampering its relocation upon the induction of differentiation [58,60]. We have detected CIT and RECQL4 in the MERCs and in the crude mitochondria, respectively; this evidence suggests that they may play a role in the function of the mitochondria, which, however, will require further studies.

Our analysis indicates a potential cooperation between these proteins for the progression of neuronal differentiation. Our data show that the structure of MERCs varies at early differentiation time points, which could be attributed to a different protein composition. Indeed, the expression levels of several MERC resident proteins decrease in parallel with proteins previously associated with NPC physiology. Taken together, our results suggest a potential functional interplay between MERCs and NPC differentiation. However, whether this is indirect, mediated by changes in the structure and activity of MERCs, or by their physical interaction remains to be determined and will be the subject of further studies.

## 5. Conclusions

Our study provides a preliminary dataset on a relatively overlooked aspect of neuronal differentiation: the changes in structure and dynamics of MERCs during differentiation. Specifically, we performed a 2D EM analysis on the well-established cellular model LUHMES, which can differentiate into dopaminergic-like neurons. Given the pivotal role of neuronal cell lines in modeling neurodegenerative diseases, this investigation opens new avenues for understanding cellular reprogramming, particularly considering how the remodeling of MERCs contributes to the metabolic and signaling adaptations essential for this process. We observed significant alterations in organelle proximity and contact site length, suggesting that MERC remodeling may play a central role in the metabolic shifts required for neuronal differentiation. Finally, we employed an omics approach that identified potential new players in MERC function, including ACAT1, PTDSS1, LMAN1, CIT, and RECQL4. These proteins, whose roles in the differentiation of neuronal precursor cells and MERC-related processes remain largely unexplored, present exciting opportunities for future research. Our 2D analysis suggests a functional interplay between MERCs and neuronal differentiation; a more complete overview could be obtained by 3D reconstruction and quantification of mitochondrial volume and density. The functional outcomes associated with any changes in MERCs also remain to be fully elucidated. In conclusion, this work paves the way for a deeper exploration of the MERC-related events that drive the transition from neural precursors to fully differentiated neurons, with potential implications for both neurodevelopment and neurodegenerative diseases.

## Figures and Tables

**Table 1:** Binomial test on cluster composition of mitochondrial genes.

Cluster	Cluster Size	Observed Mito Genes in Cluster	Expected Mito Genes in Cluster	Observed—Expected	Observed/ Expected	*p*-Value
Cluster 18	423	65	2.61 × 10^1^	3.89 × 10^1^	2.49	1.70 × 10^−11^
Cluster 2	685	8	4.22 × 10^1^	−3.42 × 10^1^	1.90 × 10^1^	1.01 × 10^−10^
Cluster 1	762	89	4.70 × 10^1^	4.20 × 10^1^	1.90	1.08 × 10^−8^
Cluster 15	455	5	2.80 × 10^1^	−2.30 × 10^1^	1.78 × 10^1^	1.37 × 10^−7^
Cluster 4	58	0	3.57	−3.57	0	5.03 × 10^−2^
Cluster 10	167	4	1.03 × 10^1^	−6.29	3.89 × 10^1^	5.06 × 10^−2^
Cluster 14	65	1	4.01	−3.01	2.50 × 10^−1^	1.89 × 10^−1^
Cluster 22	95	9	5.85	3.15	1.54	1.94 × 10^−1^
Cluster 13	33	0	2.03	−2.03	0	2.66 × 10^−1^
Cluster 5	28	0	1.73	−1.73	0	4.16 × 10^−1^
Cluster 8	34	1	2.09	−1.09	4.77 × 10^−1^	7.22 × 10^−1^
Cluster 29	36	1	2.22	−1.22	4.51 × 10^−1^	7.25 × 10^−1^
Cluster 11	37	1	2.28	−1.28	4.39 × 10^−1^	7.27 × 10^−1^
Cluster 17	38	1	2.34	−1.34	4.27 × 10^−1^	7.30 × 10^−1^

**Table 2:** Differentially expressed genes (DEGs) encoding MERC-resident proteins.

Gene	Cluster
*ACAT1*	1
*ATP2A1*	10
*DGAT2*	1
*ERO1A*	1
*HSPA9*	18
*LMAN1*	1
*PACS2*	2
*PTDSS1*	1
*PSEN2*	18
*REEP1*	2
*SNCA*	2
*TP53*	22
*ZFYVE1*	15

## Data Availability

The RNA sequencing dataset (raw reads) generated in the reported study has been deposited in the Sequence Read Archive (SRA) and is available through Bioproject #PRJNA1175585.

## Supplementary Materials

The following supporting information can be downloaded at: www.mdpi.com/article/10.3390/biom15010126/s1, Table S1: Expression browser; Table S2: GO terms; Table S3: Transcription factors and targets; Figures S1–S16: Original western blot images.
